# Turning science into teaching: a challenge for scientists

**DOI:** 10.15694/mep.2019.000007.1

**Published:** 2019-01-09

**Authors:** Simonetta Ausoni

**Affiliations:** 1 Department of Biomedical Sciences - University of Padua - Viale G. Colombo

**Keywords:** basic science, medical education, peer-reviewed learning resources, virtual lab

## Abstract

This article was migrated. The article was marked as recommended.

Teaching basic science in the medical school remains a challenge, and the lack of appropriate resources is one of important limitation. Building up such resources is difficult, time-costly and does not always result in effective, solid and student-centered instruction.

This “personal view” aims to stimulate scientists and scientific journals to engage with new ideas and innovative resources for biomedical education. The time has now come to plan research and education as mutually beneficial activities, supporting each other rather than competing with each other. Scientific research should be converted into digital learning resources hosted by scientific journals on a regular basis, and subjected to peer-review to ensure quality and integration of contents, appropriate cognitive approach and rigorous criteria of selection.

Turning science into teaching represents an investment with mutual benefits, for students and educators. Academic educators can produce resources to face the teaching burden, and gather the opportunity to increase personal productivity. Students can take advantage from being engaged in innovative learning environments where educators act as catalysts for learning, instead of just transmitters of knowledge.

## The role of basic science in the medical curriculum

The intrinsic value of basic science in medical education and clinical practice is no more questioned. Since the age of Abraham Flexner (1866-1959), who first recognized the importance to link preclinical and clinical knowledge ( Flexner, 1910), incorporation of basic science into the clinical contents has become a main goal of curriculum reforms, and has generated multiple experiments of integration at the level of programs, courses, and day-to-day activities ( Kulasegaram *et al*., 2013).

Almost a century of research and experience in the field have lent evidence that teaching basic science in the medical school provides the fundamental knowledge for medical practice, builds the appropriate context for learning, impacts on the development of inquisitive and critical minds ( Lisk *et al.,* 2017), and provides essential tools to face with complex contexts whenever clinical cases depart from the routine ( Woods *et al.,* 2007).

To best integrate basic science and clinical medicine, MD-PhD programs have been also launched in many academic institutions, with the aim to reinforce the use of basic science knowledge at the end of medical program, when students can better appreciate its relevance in practice ( Spencer *et al*., 2008).

As a matter of fact, integration of basic science and clinical contents is still a matter of debate, and a challenge. Herein, I discuss why and how scientists should invest in producing innovative resources to teach basic science in medical courses and to facilitate integration of different types of knowledge in the medical courses.

Bearing in mind that integration of basic science and clinical medicine goes beyond this level of intervention, I am nevertheless convinced that involving scientists in the production of scientifically validated educational resources could have mutual beneficial effects, for students and educators. This personal view relies on a long-lasting experience of teaching in a medical school (University of Padua, Italy) where basic science courses are grouped within the first 2-3 years of a 6-year medical program.

## Teaching basic science to medical students: a challenge for scientists

The explosion of biomedical discoveries has changed our interpretation of diseases at an unprecedented speed. Progression of nontraditional disciplines, such as bioinformatics, nanotechnology, imaging and bioengineering, has also contributed to gain insights into complex mechanisms of human function and pathologies. All scientists agree that students should be aware of the potential impact of scientific progress in medicine ( Anderson *et al*., 2011). However, translating scientific advances into teaching has become extremely complex. Main problems are how to balance contents, where to put the appropriate boundaries between essential and specialized knowledge and how to avoid the overflows of data that can challenge memory and retention, but do not provide substantial contribution to learning. On the other hand, using simplified learning tools can be harmful because they can glue the medical students to old and dated contents largely overcome by new knowledge. One of the mistakes scientists often do as educators is to arrange scientific contents keeping the same level of details as if they were in front of an audience of experts. This has two consequences. The first is the lack of a real students engagement, since activities are centered on educator’s research and scientific fame. The second is that in the context of medical courses such learning resources run the risk to be “lost in translation”, insofar as educator scientists lack practical knowledge of clinical contexts where such discoveries can be applied.

I believe that academic scientists should be in the front line to translate biomedicine, with its basic contents and new discoveries, into innovative resources for education. Scientific journals and journals of education should provide a platform to host such resources on a regular basis. There are some virtuous experiences in this field, but they have too limited impact on teaching. In 2010, to provide new opportunities for science education, PLoS Biology launched PLoS Education ( Kerfeld and Gross, 2010), an editorial series of articles and associated resources to teach life science by applying a discovery-based approach and contemporary research methods. With this smart initiative, for the first time research-based teaching activities were hosted in a research journal. Recently, PNAS offered a Teaching Resources Portal containing Core Concepts articles that explain a trending topic in a given field, and allow downloading figures, tables and podcasts for classroom discussion. In the area of clinical medicine, The New England Journal of Medicine provides an enormous repository of texts and videos of clinical cases, which can be adapted to different learning contexts.

Up-to-date, hundreds of learning tools are available on the web, but in most cases they are neither scientifically designed nor tailored to the needs of medical courses. Educators would greatly appreciate having scientifically validated learning tools as digital, easy-to-use and flexible resources. Such resources should be produced and “published” according to specific guidelines, terms and conditions. A peer-review process should ensure their quality, in terms of contents and use of cognitive science methodology. Such learning resources should include basic science knowledge and scientific advances, and should be designed to facilitate mutual links and integration with clinical cases. Innovative learning resources should also host virtual lab experiences, whose function is remarkably important in supporting scientific training of medical students. Up to date laboratory experience is lacking in many medical curricula and interest in research and translational medicine has fallen into a deep crisis ( Carnevale, 2003; Roberts et al. 2012; Waldrop, 2013).

## Investing in basic science teaching: costs, recognition and benefits

A big hole still separates science and the teaching of science in the academic environment. Some reasons concern the perception that scientists have of their role as educators. Many academic institutions do not reward good teaching and do not even invest in innovative teaching ( Anderson et al., 2011). Good teaching and good research run the risk of becoming mutually exclusive, and a largely diffused opinion is that teaching is part of the job, but does not represent a good investment for career. Studies confirm a negative association between excellence in science/clinics and the quality of academic teaching ( Marsh and Hattie, 2002).

Despite academic recognition, teaching remains a demanding mission to which many academic educators dedicate passion and big efforts. Traditional lessons and traditional books are no longer adequate to cover the continuous expansion of knowledge in biomedicine ( Schwartzstein and Roberts, 2017). Building up new learning tools is difficult, time-costly and does not always result in efficient and innovative instruction. Therefore, investing in continuous production of learning resources for biomedical education is strategic for a number of reasons. From the students’ point of view, most important advantages can be: a) quality of learning; b) integration of basic and clinical contents ( Ausiello, 2007); c) more involvement in biomedical research. From the educators’ point of view, I consider: a) the possibility to generate learning resources as academic “production”, combining expertise in research with teaching; b) the possibility to have a repository of new learning tools - a Resourceome for Biomedical Education ( Cannata *et al.,* 2005) - ready to use for building up personalized learning activities.

Finally, I want to consider one last point, which probably represents the most important aspect of this personal reflection. I mean the possibility to focus on integration of contents instead of mere contents. Having a good repository of ready-to-use teaching tools would allow teachers to focus on how to organize interactive and engaging teaching, promoting their role as mentors rather than content communicators ( Biggs, 2003). It is a widespread belief that an efficacious integration of basic and clinical science does not simply arise from placing contents in close proximity, because integration occurs within students’ mind, not in the curriculum ( Woods, 2007; Kulasegaram *et al.,* 2015; Kulasegaram *et al.,* 2017; Lisk *et al.,* 2017). Rapid access to scientifically validated learning resources should enable educators to redirect their efforts to other goals: generate the right causal relationship of basic and clinical contents; support students to view basic science knowledge from the perspective of their application ( Schwartzstein and Roberts, 2017); align curriculum and teaching methods to achieve the desired learning outcomes ( Biggs, 2003; Bandiera *et al*., 2017).

## Take Home Messages

• New learning resources are necessary to teach advanced basic science in medical courses.
• New learning resources should be generated by scientists, peer-reviewed and published on a regular basis in scientific journals.
• A repository of high quality resources for biomedical education would facilitate integration of basic and clinical contents and organization of relevant, student-centered activities.
• It’s time to link research and education as mutually beneficial, not conflictual activities.

## Notes On Contributors

Simonetta Ausoni is Assistant Professor of General Pathology at the Medical School of the University of Padua. She is a biomedical scientist in the field of Cardiovascular Research. She has been involved in evaluation and curricula design for medical programs. She is member of the Committee for Accreditation and Evaluation of the Medical Course of the University of Padua and member of the group of Medical Education in the Medical School of the University of Padua.
