# Supplementary material for: Turning science into teaching: a challenge for scientists
Source: MedEdPublish (2016). 2019 Jan 9;8:7. [Version 1] doi: 10.15694/mep.2019.000007.1 (PMC10712623; doi:10.15694/mep.2019.000007.1)
Supplement: Peer review history

# Reviewer report: Reviewer response for version 1

**DOI:** 10.21956/mep.19754.r29644

**Published:** 2019-01-26

**Authors:** Hassaan Waqar

**Affiliations:** 1 St Helens and Knowsley Teaching Hospitals NHS Trust – Lead Employer

This review has been migrated. The reviewer awarded 3 stars out of 5 An interesting opinion piece on the teaching of science at medical school. The abstract is a concise summary of the themes explored within the paper. In the introduction, the author highlights the importance of teaching basic medical science in medical school and identifies the aim of the opinion piece. The challenges that exist when scientists are tasked with teaching science to medical students are discussed and their potential solutions are explored. Some of the hurdles in the delivery of teaching science at medical school are explored, namely cost and time required to develop new tools for learning. The take home messages are appropriate and neatly summarise the potential solutions to the problems discussed in the article. Overall, the article is an aspirational piece, in which the author has presented their view on how science can be better integrated into the teaching that occurs at medical school. Perhaps this integration could be achieved through collaboration between doctors and scientists in order teach science effectively to medical students.

Reviewer Expertise:

NA

No decision status is available

# Reviewer report: Reviewer response for version 1

**DOI:** 10.21956/mep.19754.r29643

**Published:** 2019-01-22

**Authors:** Madalena Folque Patricio

**Affiliations:** 1 School of Medicine, University of Lisbon

This review has been migrated. The reviewer awarded 4 stars out of 5 It was with great interest that I read the author’s reflection on the need to make the learning of basic science relevant and meaningful. The author defends the focus on ‘integration of contents instead of mere contents’. The interest in this topic is not new: Flexner, more than a century ago was the first to ‘recognize the complementary roles of basic and clinical cycles’ and ‘integration as a challenge for medical education and all stakeholders’ was one of the recommendations of the 1988 Edinburgh Declaration just to mention two landmarks. Despite thirty years had elapsed after Edinburgh, the interest in the topic is growing to improve the current curricular integration models in practice, so far.The ‘lack of value attributed to teaching namely when compared to research’, the ‘lack of resources’ and other barriers, are reported by the author with examples on how they can be overcome to achieve curricular integration. According to him, ‘integration must be ‘a challenging priority for medical and other health care schools requesting Faculty investment’. The author’s statement ‘new learning resources should be generated by scientists, peer-reviewed and published on a regular basis in scientific journals’ may benefit from the complementary perspective proposed by Megan Anakin, a reviewer of this paper, who proposed ‘team effort as a possible alternative, namely a small resource building team consisting of academics that include a scientist, a medical practitioner, an education expert, and possibly a senior medical student’.Investing in the complementary of teaching and research is crucial if we want to see integration models to progress. The author, which last take home message stated that ‘it’s time to link research and education as mutually beneficial, not conflictual activities’ also recognizes this need.Increased collaboration, one of the four global medical education trends identified by Harden et al. in his 2018 paper ‘Analyzing Forty years of medical education through the eyes of Medical Teacher: From chrysalis to butterfly’, is also fundamental if we want integration models to progress. This is why this paper could have been entitled ‘Turning science into teaching: a challenge for scientists and teachers’, instead of just ‘Turning science into teaching: a ‘challenge for scientists’.

Reviewer Expertise:

NA

No decision status is available

# Reviewer report: Reviewer response for version 1

**DOI:** 10.21956/mep.19754.r29646

**Published:** 2019-01-10

**Authors:** P Ravi Shankar

**Affiliations:** 1 American International Medical University

This review has been migrated. The reviewer awarded 4 stars out of 5 I read with great interest the personal reflection by the author on incorporating learning of the recent advances in biomedicine into the basic sciences. I agree with the author that this presents a major challenge. I work in an offshore Caribbean medical school where the curriculum is accelerated and students complete the basic sciences within 20 months. This creates a number of challenges for students and faculty members. Integrating the basic sciences into clinical diagnosis, management and treatment is difficult. A number of unconventional areas like artificial intelligence, genomics, genetic engineering and robotics among others are playing an increasingly important role in medicine. Recognizing the appropriate level of knowledge and detail for an undergraduate medical student is an important task for an educator. In many parts of the world doctors with a basic qualification in medicine pursue advanced degrees in different basic science subjects like anatomy, physiology, pathology and pharmacology. I believe this provides advantages in understanding the clinical significance of the advances in the basic sciences and biomedicine. The MedEdPortal hosted by the Association of American Medical Colleges provides learning resources for teachers and faculty. These resources created by different medical educators are peer reviewed and freely available online.

Reviewer Expertise:

NA

No decision status is available

# Reviewer report: Reviewer response for version 1

**DOI:** 10.21956/mep.19754.r29645

**Published:** 2019-01-09

**Authors:** Megan Anakin

**Affiliations:** 1 University of Otago

This review has been migrated. The reviewer awarded 3 stars out of 5 The author presents a well-argued personal view about the ongoing challenge of how to effectively integrate basic science and clinical medicine. This challenge applies to the medical programme at my university in New Zealand because it is also structured as a six-year undergraduate degree. We also face the challenge of providing our teachers and students with relevant, up-to-date learning materials that make connections between foundation science concepts and principles, and clinically relevant applications of that scientific knowledge. The author presents well-supported ideas and evidence about why we should have well-designed learning resources for teaching basic sciences.The author’s final point about the importance of integration left me wondering about how this might be achieved. One way to address this challenge might involve a different approach than the solution offered in this paper. Rather than advocating for academic scientists to take responsibility for developing resources on their own, the author might like to consider if a team effort might be another productive way to address the need for integration. A small resource building team could consist of academics that include a scientist, a medical practitioner, an education expert, and possibly a senior medical student. This way, the academic that produces the scientific knowledge can work alongside an academic who will need to apply that knowledge in practice to provide patient care. An educational expert will help the team to focus on how the breadth and depth of that knowledge can be conveyed to students in ways that are educationally sound and promote active learning. A senior student will provide an additional perspective that can be used to fine tune the deployment and assessment of the resource. The problem of producing more resources that are “neither scientifically designed nor tailored to the needs of medical courses” will persist unless we address the process of producing resources while developing expectations and standards for the ‘content’ the resources. Thank you for making me think deeply this morning.

Reviewer Expertise:

NA

No decision status is available
